# Verhaltensbezogene und kulturelle Erkenntnisse für Bewegungsförderung bei Studierenden nutzen – das Projekt „Smart Moving“

**DOI:** 10.1007/s00103-025-04110-9

**Published:** 2025-08-12

**Authors:** Julika Loss, Julia von Sommoggy und Erdödy, Jana Rüter, Jessica Helten, Claas Christian Germelmann, Susanne Tittlbach

**Affiliations:** 1 Abteilung Epidemiologie und Gesundheitsmonitoring, Robert Koch-Institut, Gerichtstr. 27, 13347 Berlin, Deutschland; 2 Institut für Sozialmedizin und Gesundheitssystemforschung, Otto-von-Guericke-Universität Magdeburg, Magdeburg, Deutschland; 3 BaySpo – Bayreuther Zentrum für Sportwissenschaft, Universität Bayreuth, Bayreuth, Deutschland; 4 Lehrstuhl Marketing und Konsumentenverhalten, Universität Bayreuth, Bayreuth, Deutschland

**Keywords:** COM-B-Modell, Körperliche Aktivität, Gesundheitsfördernde Hochschule, Interventionen, Partizipation, COM‑B model, Physical activity, Health-promoting university, Interventions, Participation

## Abstract

**Hintergrund:**

Bewegungsmangel ist an Hochschulen (HS) weit verbreitet. Verhaltensbezogene und kulturelle Erkenntnisse („behavioural and cultural insights“, BCI) helfen, Barrieren für Bewegung bei Studierenden zu identifizieren und bedarfsgerechte Interventionen zu entwickeln. Ziel von „Smart Moving“ war, BCI zur Bewegung an 2 HS zu gewinnen und zu nutzen, um bewegungsfördernde Maßnahmen partizipativ umzusetzen.

**Methode:**

„Smart Moving“ wurde an den Universitäten Bayreuth und Regensburg von 2018 bis 2021 durchgeführt. Das Projekt wurde in 4 Schritten umgesetzt: (1) Als Zielverhalten wurde die Bewegung Studierender am Campus festgelegt, (2) es wurden Erkenntnisse zum Bewegungsverhalten gewonnen, mittels standardisierter Erhebung bei Studierenden, Photovoice und Experteninterviews, (3) an jeder HS entwickelte und implementierte eine Planungsgruppe bewegungsfördernde Maßnahmen, (4) Akzeptanz und kurzfristige Effekte ausgewählter Maßnahmen wurden in Kurzbefragungen evaluiert.

**Ergebnisse:**

Studierende saßen im Mittel 34 h pro Woche während ihres Aufenthalts am Campus. Einflussfaktoren auf das Bewegungsverhalten wurden den Kategorien Capability (kognitive/körperliche Fähigkeit), Opportunity (physische/soziale Umgebung) und Motivation zugeordnet, sie umfassten z. B. mangelnde Kenntnis über Zugänge, schlechte Erreichbarkeit von Bewegungsangeboten, vorherrschende Norm, dass im Sitzen gelernt wird, sowie Scham beim Bewegen vor anderen. Es wurden verschiedene Ansätze zur Bewegungsförderung entwickelt: Bewegungspausen in Lehrveranstaltungen, aktivierende Möbel mit Sitz‑/Stehmöglichkeit, Bewegungshinweise im Außengelände, motivierende Bewegungsanreize. Die Maßnahmen stießen auf hohe Akzeptanz unter Studierenden.

**Diskussion:**

Die gewonnenen BCI halfen, bedarfsgerechte Bewegungsförderung an HS umzusetzen. Weitere Studien müssen langfristige Auswirkungen auf Bewegungsverhalten untersuchen.

## Abstract (en)

**Background:**

Physical inactivity is widespread at universities. To promote physical activity among students, it is important to understand their needs. Behavioral and cultural insights (BCIs) help to identify barriers to physical activity and to develop appropriate interventions. The aim of “Smart Moving” was to use BCIs to implement measures to promote physical activity in two universities.

**Method:**

“Smart Moving” was carried out at the universities of Bayreuth and Regensburg between 2018 and 2021. The project was implemented in four steps: (1) the target behavior was defined as students being physically active on campus; (2) knowledge about physical activity behavior was gained using a standardized survey of students, photo voice, and expert interviews; (3) a planning group at each university developed and implemented measures to promote physical activity; and (4) acceptance and short-term effects of selected measures were evaluated in short surveys.

**Results:**

University students spent an average of 34 h per week sitting during their stay on campus. Factors influencing physical activity were assigned to the following categories: capability (cognitive/physical ability), opportunity (physical/social environment), and motivation. These included, for example, a lack of knowledge about access, poor accessibility of exercise opportunities, the prevailing norm that learning involves sitting, and shame when exercising in front of others. Various approaches to promote physical activity were developed: movement breaks in lectures, activating desk furniture with sitting/standing options, movement instructions in the outdoor area, and motivational interventions for exercise. The measures were well received by students.

**Discussion:**

The BCI data helped implement needs-based physical activity promotion at universities. Further studies are needed to investigate the long-term effects on physical activity behavior.

## Hintergrund

Bewegungsmangel und sitzendes Verhalten gelten als relevante Gesundheitsrisiken. Körperliche Inaktivität geht mit einem erhöhten Risiko für die Gesamtmortalität sowie für verschiedene chronische Erkrankungen einher, z. B. Diabetes, verschiedene Krebsarten oder Herz-Kreislauf-Erkrankungen [1, 2]. Für lang anhaltende Sitzzeiten ist ein Zusammenhang mit körperlichen Einschränkungen, niedrigerer gesundheitsbezogener Lebensqualität und Depression belegt [3].

Deutschland gehört zu den Ländern mit einer relativ hohen Sitzdauer [4]. Besonders lange Sitzzeiten haben junge Erwachsene (18- bis 29-Jährige) der oberen Bildungsgruppe [4, 5]. Nur 65 % dieser Altersgruppe in Deutschland bewegen sich pro Woche 150 min oder mehr, wie von der Weltgesundheitsorganisation (WHO) empfohlen [6]. Daher ist es sinnvoll, Maßnahmen zur Förderung von Bewegung und Verringerung von Sitzzeiten in der Lebenswelt von jungen Erwachsenen umzusetzen, beispielsweise in Hochschulen.

An Hochschulen ist körperliche Inaktivität weit verbreitet und in typischen Abläufen und Strukturen verankert: Studierende sitzen während der Lehrveranstaltung oder beim Lernen in der Bibliothek, auch Pausen werden mit Sitzen verbracht (z. B. beim Essen). Maßnahmen der Bewegungs- und Gesundheitsförderung bei Studierenden haben zudem einen besonderen Wert, da Studierende als Multiplikatorinnen und Multiplikatoren wirken können, insbesondere wenn sie im weiteren Lebensverlauf zu Führungskräften bzw. Entscheidungsträgerinnen und Entscheidungsträgern werden. So können Erfahrungen mit und Erfolge von Bewegungsförderung an Hochschulen auch später in andere gesellschaftliche Bereiche hineingetragen werden [7].

Für die Planung von bewegungsfördernden Maßnahmen kann es sinnvoll sein, eine verhaltenswissenschaftliche Perspektive einzunehmen [8]. Die Entwicklung von Interventionen orientiert sich dabei an Erkenntnissen zu Barrieren bzw. Förderfaktoren von Bewegungsverhalten. Die WHO verwendet für derartige Erkenntnisse den Begriff der verhaltensbezogenen und kulturellen Erkenntnissen (Behavioural and Cultural Insights, BCI) und empfiehlt, sich für Interventionen zur Verhaltensänderung an dem theoretischen Modell des „Behaviour Change Wheel“ von Susan Michie [9] zu orientieren [10]. Aufbauend auf den identifizierten Verhaltensdeterminanten werden Interventionen entwickelt, die Menschen unterstützen sollen, das entsprechende Verhalten in gesundheitsförderlichem Sinne zu verändern. Interventionstypen können dabei von Befähigung, Umstrukturierung der Umgebung, kommunikativen Maßnahmen bis hin zu Änderungen von Regeln reichen [11]. Empfohlen wird darüber hinaus die Einbindung von Stakeholdern bzw. Betroffenen in die Planung der Maßnahme [10, 12]. Schließlich muss die Intervention umgesetzt, verbreitet und evaluiert werden [11].

Einige Studien haben bereits bewegungsfördernde Maßnahmen explizit basierend auf BCI entwickelt. Beispiele umfassen eine Intervention zu gesunden Lebensstilen bei Menschen mit Diabetes mellitus [13], eine Studie zur Förderung mentaler Gesundheit über sportliche Betätigung bei Studierenden [14] oder die Entwicklung von Ansätzen zur Reduzierung von Sitzzeiten im Büro [15].

Auch das Projekt „Smart Moving“ nutzte BCI, um Bewegung im Setting Hochschule zu erhöhen und sitzendes Verhalten zu verringern. Angesprochen wurde dabei die Gruppe der Studierenden. „Smart Moving“ wurde in Kooperation zwischen zwei Universitäten, dem bayerischen Kompetenzzentrum für Ernährung sowie der Techniker Krankenkasse entwickelt. Studierende und Mitarbeitende entwickelten in einem partizipativen Prozess verschiedene Maßnahmen zur Bewegungsförderung, die an die Bedürfnisse der jeweiligen Hochschule angepasst waren. Als Grundlage wurden zuvor Erkenntnisse dazu gewonnen, welche Faktoren am Campus Aktivitätsverhalten im Alltag verhindern bzw. fördern.

Einzelne Elemente des Projekts „Smart Moving“ wurden bereits separat publiziert, z. B. die Analyse mittels Photovoice [16], die Evaluation von bewegungsfördernden Bibliotheksarbeitsplätzen [17] oder die Rolle verschiedener Einflussfaktoren [18]. Dieser Artikel erläutert den gesamten Planungs‑, Umsetzungs- und Evaluationsprozess von „Smart Moving“, in Anlehnung an den BCI-Ansatz der WHO.

## Methodik

„Smart Moving“ wurde von 2018–2021 an den Universitäten Bayreuth (UBT) und Regensburg (UR) umgesetzt. Die Planung und Umsetzung erfolgten in 4 Schritten, die sich dem Vorgehen „Tailoring Health Programmes“ zuordnen lassen, das die WHO für den BCI-Ansatz vorschlägt [10, 19].

### 1. Analyse der Situation: Problemerkennung und Identifikation relevanter Bevölkerungsgruppen und Verhaltensweisen

Während der Rahmen des Projekts feststand (Bewegungsförderung an Hochschulen), wählten die Kooperationspartner in mehreren Sitzungen die konkrete Zielgruppe aus und präzisierten das Verhalten, dessen Veränderung erreicht werden sollte. Für die Situationsanalyse flossen zudem Ergebnisse einer standardisierten Erhebung des Bewegungs- und Sitzverhaltens in Anlehnung an Fuchs et al. [20] und Marshall et al. [21] unter Studierenden der beiden Universitäten ein, die 2018 zu Beginn von „Smart Moving“ durchgeführt wurde. Außerdem wurden wahrgenommene Barrieren und Bedürfnisse zu Aktivität erfasst [22]. Es wurden Angaben von *n* = 827 Studierenden verschiedener Studiengänge (UBT: *n* = 512, UR: *n* = 315, weiblich (f) = 59 %) erhoben.

### 2. Forschung: Erkenntnisgewinn über Barrieren und Einflussfaktoren auf das Verhalten in der relevanten Bevölkerungsgruppe

Um Erkenntnisse zu den lokalen Barrieren, Potenzialen und Bedarfen im Hinblick auf Alltagsbewegung zu gewinnen, wurden in beiden Universitäten jeweils semistandardisierte Experteninterviews sowie Photovoice-Projekte mit Studierenden durchgeführt. Die semistandardisierten *Experteninterviews* wurden mit Lehrenden, Studierenden sowie Beschäftigten des Sportzentrums und der Verwaltung geführt (UR: *n* = 10 bzw. UBT: *n* = 5). Tab. 1 zeigt die Leitfragen der Interviews.

**Tab. 1:** Interviewleitfaden zur Alltagsbewegung von Studierenden für Experteninterviews, die im Rahmen des Projektes „Smart Moving“ an den Universitäten Bayreuth und Regensburg durchgeführt wurden (Laufzeit 2018–2021)

Interviewleitfaden für Lehrende, Studierende und Beschäftigte der jeweiligen Hochschule
1. Wenn Sie an den Alltag von Studierenden auf dem Campus denken: Wie schätzen Sie die Möglichkeiten zur Alltagsbewegung an der Universität ein? (Im Allgemeinen, in den Veranstaltungen, in den Pausen …)
2. Was sind Faktoren, die die Alltagsbewegung von Studierenden auf dem Campus fördern? (Inwiefern? In welchem Ausmaß?)
3. Was sind Ihrer Meinung nach Hemmnisse für Alltagsbewegung von Studierenden? Welche Faktoren hindern Studierende daran, sich im Uni-Alltag mehr zu bewegen?
4. Bei Mitarbeitenden: Was ist Ihr Eindruck – wo und wann sind Studierende in Bewegung? Wo sind Studierende überhaupt nicht in Bewegung? Welche Unterschiede gibt es zwischen Semesterzeiten und vorlesungsfreier Zeit? Welche Unterschiede gibt es zwischen den Jahreszeiten? (Vorzeigen einer Campus-Map)
5. Was könnte/müsste verändert werden, damit sich die Möglichkeiten für Studierende verbessern, sich im Alltag an der Universität mehr zu bewegen?
6. Gibt es noch etwas, dass Sie dem Gespräch hinzufügen möchten? Vielleicht ein wichtiger Aspekt, der bisher gar nicht erwähnt wurde?

Für den *Photovoice-Ansatz* wurden 22 (UBT) bzw. 24 (UR) Studierende pro Standort rekrutiert (jeweils *n* = 14 f), um mit ihren Smartphones mindestens 15 Fotos von Orten und/oder Situationen zu machen, in denen Bewegung im Alltag als leicht oder schwer möglich empfunden wurde. In anschließenden moderierten Fokusgruppen (*n* = 5 pro Universität, je 3–5 Teilnehmende) wurden die ausgedruckten Fotos vorgelegt. Die Studierenden wurden gebeten zu erklären, was in der fotografierten Szene zu sehen ist [23] und wie das Bild mit körperlicher Aktivität zusammenhängt, d. h. inwiefern Bewegung gefördert oder verhindert bzw. erschwert wird (siehe auch [16]).

Die Experteninterviews sowie die Fokusgruppendiskussionen wurden mittels Inhaltsanalyse ausgewertet [24]. Die identifizierten Einflussfaktoren wurden basierend auf dem von der WHO für BCI empfohlenen COM-B-Modell kategorisiert [25]. Die Abkürzung steht für „capability, opportunity and motivation for behaviour“. Dieses Modell postuliert, dass ein Gesundheitsverhalten (Behaviour) nur dann auftritt, wenn die Menschen die Fähigkeiten (Capabilities) und Gelegenheit (Opportunity) haben, dieses Verhalten auszuführen, und motiviert sind, entsprechende Entscheidungen zu treffen (Motivation). Fähigkeit bezieht sich z. B. auf individuelle Gesundheitskompetenz oder körperliche Fähigkeiten wie Fitness; Gelegenheiten umfassen die unterstützenden Rahmenbedingungen im Lebensumfeld, z. B. leichter Zugang, soziale Normen oder organisationale Gesundheitskompetenz. Unter Motivation werden in diesem Modell sowohl reflektierte, bewusste Verhaltensabsichten als auch intuitive Prozesse wie Gewohnheiten und Gefühle verstanden [25].

### 3. Entwicklung (komplexer) Intervention, aufbauend auf verhaltensbezogenen und kulturellen Erkenntnissen; Einbindung von Stakeholdern

An beiden Standorten wurde je eine Planungsgruppe etabliert und moderiert, die sich von 11/2018 bis 01/2020 regelmäßig getroffen hat (*n* = 10 an UR bzw. *n* = 8 Treffen an UBT). Die Gruppen setzten sich zusammen aus Studierenden, Lehrenden, Mitarbeitenden aus Wissenschaft und Hochschulverwaltung, Bibliothek und Sportzentrum. An der UBT gab es bereits vor „Smart Moving“ ein universitäres Gesundheitsmanagement, an das die Planungsgruppe für „Smart Moving“ angegliedert wurde. Es wurden zunächst die gewonnenen verhaltensbezogenen Erkenntnisse berichtet, darauf aufbauend wurden Ideen für Interventionen gesammelt, priorisiert und schrittweise in die Umsetzung gebracht. Für die Konkretisierung und Realisierung einzelner bewegungsförderlicher Maßnahmen wurden teilweise Untergruppen gebildet.

### 4. Monitoring und Evaluation der Maßnahmen

Für die Maßnahmen wurden, soweit zeitlich umsetzbar, orientierende Erhebungen unter Studierenden geplant und umgesetzt, die sich auf Nutzung und Akzeptanz der Maßnahme und deren eingeschätzten Einfluss auf Verhaltensänderungen bezogen. Hierfür wurden kurze standardisierte Fragebögen entwickelt, pilotiert, überarbeitet und je nach Maßnahme bei unterschiedlichen Stichproben von Studierenden eingesetzt.

Der Fragebogen, mit dem die Akzeptanz einer neu eingeführten Bewegungspause erhoben wurde, enthielt beispielsweise folgende standardisierte Fragen bzw. Aussagen, denen zugestimmt bzw. die abgelehnt werden konnten: „Wie oft haben Sie die ‚Bewegte Pause‘ mitgemacht?“ „Die Übungen wurden verständlich vorgemacht.“ „Ich konnte alle Übungen mitmachen.“ „Die Übungen haben mir Spaß gemacht.“ „Die Dauer des Videos ist angemessen.“ „Die ‚Bewegte Pause‘ hat mir insgesamt gut gefallen.“ Und: „Wie oft soll die ‚Bewegte Pause‘ in Lehrveranstaltungen angeboten werden?“ Eine Erhebung zur Akzeptanz von motivierenden Wandstickern enthielt u. a. folgende standardisierten sowie offenen Fragen: „Ist dir der Schriftzug aufgefallen?“ „Hat dich der Schriftzug angeregt, die Treppe zu nehmen?“ „Wie findest du die Aktion?“

Für die Evaluation von neu eingerichteten bewegungsfördernden Arbeitsplätzen in der Bibliothek wurden Leitfadeninterviews geführt. Diese umfassten die Themen „Akzeptanz und Bewertung“ (z. B. „Inwiefern möchten Sie die bewegungsfördernden Arbeitsplätze weiterhin nutzen?“), „Auswirkungen auf Arbeitsweise“ (z. B. „Inwiefern haben Sie nach der Woche an den bewegungsfördernden Arbeitsplätzen Veränderungen in Ihrer Arbeitsweise feststellen können?“) sowie „Auswirkungen auf physische und psychische Gesundheit“ (z. B. „Inwiefern hatte die Nutzung der bewegungsfördernden Arbeitsplätze Auswirkungen auf Ihre Gesundheit?“). Zudem erfolgten semistandardisierte Interviews mit den Teilnehmenden der Planungsgruppen; sie sollten die umgesetzten Maßnahmen beurteilen und die wichtigste Veränderung benennen, die im Rahmen von „Smart Moving“ bewirkt wurde.

Zur Analyse der Veränderung des Bewegungsverhaltens war für 2020 eine erneute Erhebung des Bewegungs- und Sitzverhaltens unter Studierenden vorgesehen. Aufgrund der Beschränkungen der Covid-19-Pandemie musste sie auf die Home-Learning-Situation angepasst werden und konnte nicht zur Beurteilung der Verhaltensänderung durch „Smart Moving“ herangezogen werden.

## Ergebnisse

### 1. Analyse der Situation: Problemerkennung und Identifikation relevanter Bevölkerungsgruppen und Verhaltensweisen

Die Kooperationspartner verständigten sich darauf, die Maßnahmen mit dem Fokus auf Studierende zu planen, um Barrieren und Förderfaktoren im Alltag spezifisch zu erfassen und zu adressieren. Eine Ausweitung auf die Mitarbeitenden wurde als mögliches Folgeprojekt angedacht. Zudem wurde das Verhalten genauer definiert: Es sollte um Alltagsbewegung, Sporttreiben und Sitzen an der Hochschule sowie auf dem Weg zur bzw. von der Hochschule gehen. Das Bewegungsverhalten unabhängig vom Studierendenalltag, z. B. in der Freizeit, sollte nicht adressiert werden.

Die Erhebung ergab, dass Studierende während ihres Aufenthalts am Campus im Mittel 34 h in der Woche sitzen (zusätzlich zu den Sitzzeiten zu Hause, in der Freizeit oder beim Transport), insbesondere während Lehrveranstaltungen (17 Std/Woche), in der Bibliothek (10 Std/Woche) und in der Mensa bzw. Cafeteria (4 Std/Woche), und dass sie das Sitzen an der Hochschule selten unterbrechen. Niedrig intensive Alltagsbewegungen, z. B. Laufen von einer Lehrveranstaltung zur Mensa, sowie aktiver Transport von/zur Hochschule, betrugen 6 h pro Woche. Grundsätzlich war das Sitzverhalten an beiden Universitäten und zwischen den Geschlechtern ähnlich, wobei Frauen tendenziell länger saßen als Männer und jüngere länger als ältere Studierende.

### 2. Forschung: Erkenntnisgewinn über Barrieren und Einflussfaktoren auf das Verhalten in der relevanten Bevölkerungsgruppe

Die Experteninterviews sowie die Photovoice-Erhebung konnten eine Vielzahl an Faktoren am Campus sowie im studentischen Alltag identifizieren, die Bewegungsverhalten beeinflussen. Zudem wurden in der Erhebung zu Beginn des Projekts Barrieren für mehr Bewegung am Campus abgefragt. Die Faktoren wurden zusammengefasst und gemäß COM-B-Modell den Kategorien „Capability“, „Opportunity“ und „Motivation“ zugeordnet. Die Ergebnisse zeigt Tab. 2. Einflussfaktoren, die sich auf strukturelle Eigenschaften des Außengeländes bezogen, wurden dabei am häufigsten genannt. Eine ausführliche Beschreibung der Photovoice-Studie findet sich bei [16].

**Tab. 2:** Erkenntnisse aus Experteninterviews (*n* = 15), Photovoice-Erhebungen (*n* = 46) sowie einer standardisierten Erhebung unter Studierenden (*n* = 827) zu den Einflussfaktoren auf das Bewegungsverhalten von Studierenden an den Universitäten Bayreuth und Regensburg (Projekt „Smart Moving“, Laufzeit 2018–2021). Zusammenfassung gemäß COM-B-Modell („capability, opportunity and motivation for behaviour“)

Capability	Opportunity		Motivation
**Kognitive und körperliche Fähigkeit (Kenntnisse, Fitness)** *Barrieren*: – Information über Sportangebote an Hochschulen und Zugangsberechtigung sind eingeschränkt – Es ist unklar, welche Bereiche der Sportanlagen am Campus kostenfrei genutzt werden dürfen – Es ist unklar, welche sportlichen Tätigkeiten auf Grünflächen erlaubt bzw. verboten sind, z. B. (Ball‑)Spiele	**Physische Umgebung (Strukturen, Angebote, Zugang, Attraktivität)** *Förderfaktoren*: – Campus ist mit attraktiven Fahrradwegen gut erreichbar – Rundwege und Fußwege im Grünen laden zu Spaziergängen ein – Breite, offene Treppen motivieren (unbewusst) zu deren Nutzung *Barrieren*: – Mangel an (überdachten) Fahrradständern und an Stauraum für Helme/Regenkleidung erschwert aktiven Transport – Treppen im Außengelände behindern Fahrradfahren auf dem Campus, Umgehungswege sind nicht ausgeschildert – Es fehlen Bewegungsangebote für Alltagsbewegung, z. B. ausleihbare Sport‑/Spielgeräte oder aktive Pausengestaltung – Outdoor-Geräte (Slackline, Boulderturm) befinden sich am Rand des Campus und sind nicht kurzfristig erreichbar – Treppenhäuser sind oft versteckt oder weniger zugänglich als Aufzug	**Sozialer Kontext, soziale Erwartungen und Normen** *Barrieren*: – Es herrscht das Grundverständnis vor, dass Lernen im Sitzen geschehen müsse – Pausen zwischen Lehrveranstaltungen sind sehr kurz – Sitzendes Verhalten in Bibliotheken und Vorlesungsräumen wird als unvermeidlich gesehen	**Einstellungen, Risikobewertung, Emotionen, Zuversicht** *Barrieren*: – Grünflächen motivieren eher zum Hinsetzen und Entspannen, Bewegungsanreize fehlen – In der Bibliothek wird für Bewegungspausen nicht aufgestanden, aus Angst, Unterlagen und Rechner ungeschützt am Platz zurückzulassen – Scham verhindert die Nutzung von Bewegungsgeräten am Sportgelände aus Sorge, von „fitten“ Sportstudierenden beobachtet zu werden – Fehlende Selbstwirksamkeit

### 3. Entwicklung (komplexer) Intervention, aufbauend auf verhaltensbezogenen und sozialwissenschaftlichen Erkenntnissen; Einbindung von Stakeholdern

**Abb. 1:**
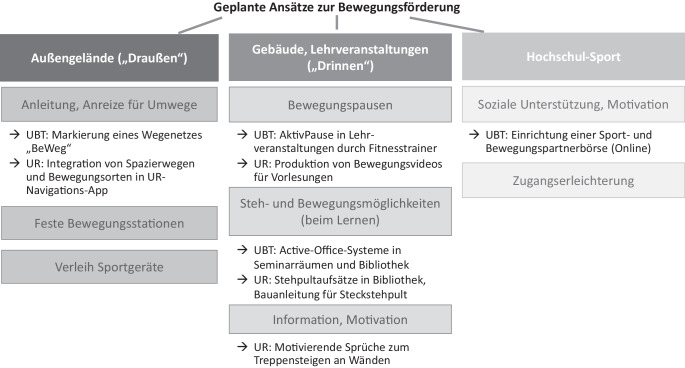
Von der Planungsgruppe identifizierte Handlungsansätze zur Bewegungsförderung von Studierenden an den Universitäten Bayreuth (UBT) und Regensburg (UR) im Rahmen des Projekts „Smart Moving“ (Laufzeit 2018–2021). *Nicht alle Ansätze konnten während der Projektlaufzeit umgesetzt werden; die Auflistung unter den Kästen zeigt, welche konkreten Interventionen an den jeweiligen Universitäten erfolgreich realisiert wurden*

Aufbauend auf den verhaltensbezogenen Erkenntnissen entwickelten die Planungsgruppen für ihre jeweilige Hochschule Maßnahmen zur Erleichterung und Förderung von Bewegung von Studierenden. Dabei wurden zunächst Handlungsansätze im Sinne von übergeordneten Interventionskategorien wie „Bewegungspausen“ oder „Anreize für Umwege“ identifiziert (Abb. 1). Ausgehend von diesen Handlungsansätzen wurden konkrete einzelne Interventionen bzw. Maßnahmen geplant. Insgesamt wurden 8 bewegungsfördernde Maßnahmen entwickelt (4 je Hochschule) und größtenteils umgesetzt. Für die Umsetzung der einzelnen Ideen wurden zum Teil weitere Akteurinnen und Akteure einbezogen, z. B. die Bibliotheksleitung, eine Krankenversicherung oder eine Werbeagentur.

Folgende Interventionen wurden umgesetzt:

#### Bewegungspausen.

Die Planungsgruppe an der UR entwickelte, drehte und vertonte drei Versionen eines 5‑minütigen *Videoclips* (https://mediathek2.uni-regensburg.de/list/1505), in dem zwei junge Personen Bewegungsübungen in der Sitzreihe eines Hörsaals vormachen. Studierende der Sport- und der Medienwissenschaften unterstützten bei der Auswahl der Übungen und technischen Umsetzung. Die Videos können von Dozierenden während der Lehreinheit aufgerufen werden.

An der UBT wurde eine *AktivPause* eingeführt, die in Lehrveranstaltungen vor Ort und digital stattfindet. Dabei werden Lockerungs- und Kräftigungsübungen von Fitnesstrainern des universitären Gesundheitsmanagements vorgeführt. Während der Covid 19-Pandemie wurde die *AktivPause* speziell für das Home Learning umgestaltet.

#### Anleitung und Anreize für Umwege.

Für die UR gab es bereits die Smartphone-App „UR Walking“ als Navigationshilfe für den Campus. Zusammen mit den Betreibern wurden verschiedene Aspekte in die App integriert, um Alltagsbewegung zu fördern. So kann man nun zu verschiedenen „Bewegungsorten“ navigieren, z. B. botanischem Garten oder Slackline-Park. Zudem wurden drei Spazierwege und eine Laufstrecke rund um den Campus in die App implementiert, die sich z. B. für eine 30-minütige Pause zwischen Veranstaltungen eignen. Weiterhin wurde ein mit Schildern ausgezeichneter Aktivpfad mit unterschiedlichen sportlichen Übungen rund um die UR erstellt.

An der UBT wurde das Wegenetz des Campus unter dem Schlagwort „BeWeg“ grafisch aufbereitet, um Bewegungsanreize auf den Uni-Wegen zu schaffen. Bunte Markierungen auf dem Boden verdeutlichen die Anzahl der zurückgelegten Schritte, ausgehend vom Ausgangspunkt, einem BeWeg-Orientierungsschild an der zentral gelegenen Mensa. Die Zeichen sollen zur Reflexion über die eigenen Bewegungsmuster anregen und motivieren, mehr Schritte zu gehen. Zudem wurde BeWeg mit einem bereits bestehenden „CampusAktivPfad“, eine Art Trimm-dich-Pfad, zusammengelegt.

#### Information und Motivation.

In drei Gebäuden wurden an der UR in der Nähe von Treppen und Aufzügen mittels großflächiger Sticker humorvolle Sprüche an den Wänden angebracht, um zum Treppensteigen zu motivieren. Hierdurch soll auf die (oftmals versteckte) Treppe aufmerksam gemacht werden.

An der UBT wurde eine *Sport- und Bewegungspartnerbörse* als Angebot auf der Online-Moodle-Plattform eingerichtet. Auf dieser Sport- und Bewegungspartner-Plattform können sich Trainings- und Bewegungspartner und -partnerinnen suchen und finden.

#### Bewegte Sitz- und Stehmöglichkeiten.

Durch die Teilnahme einer Bibliotheksmitarbeiterin an der Planungsgruppe an der UR entstand eine Verbindung zum Arbeitskreis „Lernraumkonzepte“ der Bibliothek, die einen Leitfaden für künftige Lernräume entwickelte. Diese Leitlinie gilt für die nächsten 5–10 Jahre und sieht nun in jedem neu gestalteten Lernraum ein Element zur Bewegungsförderung vor. Zusätzlich schaffte die Bibliothek, motiviert durch die Aktivitäten der Planungsgruppe, ergonomische Möbel zum Arbeiten im Stehen an und stellt Stehpultaufsätze für die Tische zur Verfügung. Weiterhin haben zwei Studentinnen eine Anleitung für den Bau eines Steckstehpultaufsatzes aus Holz erstellt. Ein geplanter Workshop in der Holzwerkstatt zum Thema „Stehpulte bauen“ konnte pandemiebedingt nicht angeboten werden.

**Abb. 2:**
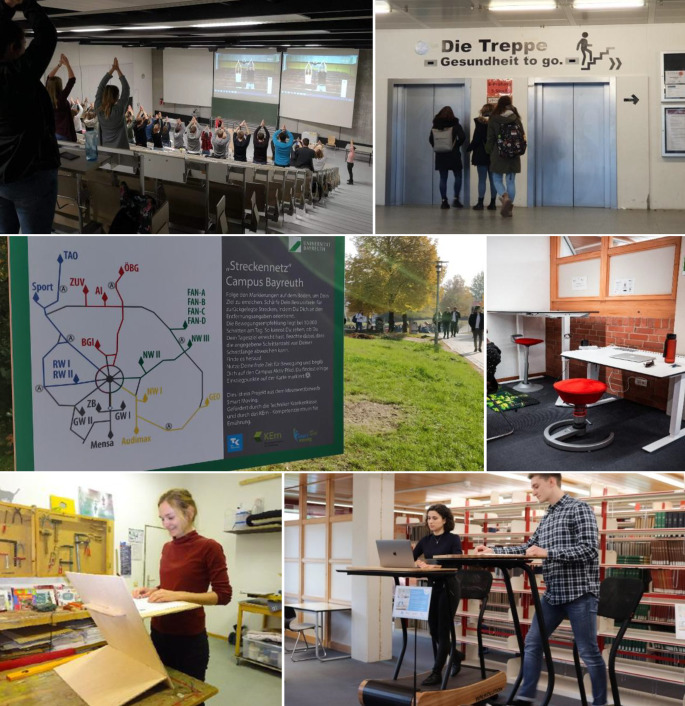
Beispiele der umgesetzten Maßnahmen zur Bewegungsförderung von Studierenden an den Universitäten Bayreuth (UBT) und Regensburg (UR) im Rahmen des Projekts „Smart Moving“ (Laufzeit 2018–2021). *Legende: von oben links: *Einsatz der Videos zur Aktiven Pause während einer Lehrveranstaltung (UR); motivierende Sticker mit Hinweisen zum Treppensteigen (UR); Ausschilderung von Laufwegen mit Ermutigung zu Spaziergängen (BeWeg, UBT); Active-Office-Systeme in der Bibliothek (UBT); selbstgebauter Stehpultaufsatz, für den eine Bauanleitung entwickelt wurde (UR); Lernlaufbänder (UBT). Das Bild *unten* *rechts* mit Genehmigung von Universität Bayreuth/Smart Moving/KErn/Max Dörres

An der UBT wurden sogenannte *Active-Office-Systeme *als aktivierende Studiermöbel in Seminarräumen und der Bibliothek aufgestellt, um studentische Arbeitsplätze bewegungsfreundlicher zu gestalten und einen Wechsel zwischen Sitzen und Stehen zu ermöglichen. Weiterhin wurden als Beitrag zu „Smart Moving“ zwei Lernlaufbänder angeschafft und in der Bibliothek platziert. Abb. 2 zeigt Fotos ausgewählter Maßnahmen.

Beide Planungsgruppen sammelten Vorschläge für feste Bewegungsstationen im Außengelände sowie in den Wandelhallen und planten ein Ausleihsystem für Sport- und Spielgeräte. Pandemiebedingt konnten vereinzelte Ideen nicht weiterverfolgt werden, wurden jedoch zu einem späteren Zeitpunkt an der UBT erneut aufgegriffen [26].

### 4. Monitoring und Evaluation der Maßnahmen

Die bewegungsfördernden Maßnahmen wurden hinsichtlich ihrer Wahrnehmung, Nutzung und Wirkung im standardisierten Online-Fragebogen abgefragt. Von allen Maßnahmen waren die *Active-Office-Systeme* (UBT) und die *Wandsticker zum Treppensteigen* (UR) am besten bekannt und wurden am häufigsten als Bewegungsanreiz genutzt. Den Studierenden wurde zudem die Frage gestellt, ob die Maßnahmen zu mehr Alltagsbewegung und kürzeren Sitzphasen beitragen. Auch hier wurde die Wirkung der *Active Offices* mit 44 % an der UBT und der *Treppen-Nudges* mit 34 % an der UR am positivsten eingeschätzt.

Zudem wurden ausgewählte Einzelmaßnahmen evaluiert. Für die Bewertung der Bewegungsvideos (UR) wurde eine standardisierte Erhebung unter 168 Studierenden (f: 68 %, Alter: 22 ± 3 Jahre) durchgeführt. Sie hatten im Mittel 2‑mal an der „Bewegten Pause“ teilgenommen und bewerteten sie insgesamt sehr positiv: Sie fanden sie verständlich, die Übungen haben der großen Mehrheit Spaß gemacht und wurden als positiv für die Gesundheit eingestuft. Die überwiegende Anzahl wünscht sich regelmäßig eine „Bewegte Pause“, d. h. einmal (38 %) oder mehrmals am Tag (19 %) oder sogar in jeder Lehrveranstaltung (28 %).

Zudem wurden 97 Personen vor Ort an den entsprechenden Fahrstühlen mittels eines kurzen Fragebogens zu den *Wandstickern* (UR) befragt. Der überwiegenden Mehrheit war der Schriftzug aufgefallen. 5 % gaben an, wegen des Schriftzugs an diesem Tag die Treppe genommen zu haben, weitere 6 % wurden dazu angeregt, darüber nachzudenken, die Treppe zu nehmen. 81 % der Studierenden kreuzten an, ohnehin oft die Treppe zu nehmen. In den Freitextantworten wurde die Aktion als gesundheitsförderlich gelobt; dabei sei sie nicht zu aufdringlich, sondern anregend.

Zusätzlich wurde eine semistandardisierte Befragung zur Akzeptanz und Bewertung der *Active-Office-Systeme* bei Nutzern und Nutzerinnen (*n* = 4) durchgeführt [17]. Die Ergebnisse der Leitfadeninterviews zeigten eine hohe Akzeptanz und positive Bewertung. Die Mischung aus Stehen und Sitzen habe den Studierenden zu mehr Bewegung und zu längeren Konzentrationsphasen verholfen.

Die Teilnehmenden der Planungsgruppen begrüßten das Format und das Vorgehen der gemeinsamen Arbeit. Allerdings wurde kritisiert, dass sie sich ehrenamtlich für die Projektumsetzung einsetzten und die finanziellen Mittel nicht reichten, um viele Ideen umzusetzen. Die umgesetzten Maßnahmen wurden von fast allen Interviewten überwiegend sehr positiv bewertet, aber auch teilweise als zeitaufwendig beschrieben, zum Beispiel die Erstellung der Bewegungsvideos und Planung von weiteren Maßnahmen zu deren Verbreitung. Als wichtigste Veränderung durch „Smart Moving“ wurde oft genannt, dass das Thema Bewegungsverhalten – auch während des Lernens – mehr Sichtbarkeit bekommen hat und dadurch das Bewusstsein zur Bedeutung dieses Themas deutlich gestiegen sei.

Eine Übersicht über alle Phasen des Projektes und der entsprechenden methodischen Ansätze und gewonnen Erkenntnisse bzw. Ergebnisse zeigt Tab. 3.

**Tab. 3:** Übersicht über alle Phasen des Projektes „Smart Moving“ an den Universitäten Bayreuth und Regensburg, Laufzeit 2018–2021. In Anlehnung an das Vorgehen der Weltgesundheitsorganisation, Regionalbüro Europa, für Interventionen auf Basis von verhaltensbezogenen und kulturellen Erkenntnissen („behavioural and cultural insights“, BCI; [10])

Phasen für Anwendung des BCI-Ansatzes	Methodische Umsetzung im Projekt „Smart Moving“	Erfolgte Schritte und Ergebnisse
**Analyse der Situation** Problemerkennung und Identifikation relevanter Bevölkerungsgruppen und Verhaltensweisen	– Präzisierung von Zielverhalten und konkreten Bevölkerungsgruppen – Standardisierte Erhebung zum Bewegungs- und Sitzverhalten von Studierenden	*Festlegung auf…* – Thema: Bewegungs- und Sitzverhalten während des Aufenthaltes an der Hochschule sowie aktiver Transport zur/von der Hochschule – Zielgruppe: Studierende
**Forschung** Erkenntnisgewinn über Barrieren und Einflussfaktoren auf das Verhalten in der relevanten Bevölkerungsgruppe	Semistandardisierte Experteninterviews mit Hochschulmitarbeitenden und Studierenden Photovoice-Projekte mit Studierenden Standardisierte Erhebung zu Barrieren und Bedürfnissen	*Barrieren im Bereich …* – der Strukturen („opportunities“) betreffen u. a. fehlende Bewegungsanreize und Sportmöglichkeiten auf den Grünflächen sowie Grundverständnis, dass Lernen im Sitzen geschieht – der Fähigkeiten („capabilities“) betreffen z. B. fehlendes Wissen über Nutzungsmöglichkeiten von Grünflächen – der Motivation betreffen v. a. Scham bei Bewegung in der Öffentlichkeit
**Intervention** Entwicklung (komplexer) Maßnahmen mit Stakeholdern, aufbauend auf gewonnenen verhaltensbezogenen Erkenntnissen	Etablierung einer Planungsgruppe (je Hochschule), regelmäßige Treffen, Identifizierung und Priorisierung von Ansätzen, konkrete Umsetzung von Maßnahmen Einbindung von verschiedenen Bereichen der Hochschule, Teilnehmende: u. a. Lehrende, Studierende, Verwaltungsangestellte, Mitarbeitende des Sportzentrums und der Bibliothek	*Es wurde eine Reihe von Interventionen in den Hochschulen umgesetzt, u.* *a.* – Etablierung von Aktiven Pausen in Lehrveranstaltungen (durch Fitnesstrainer oder neu entwickelte Videos) – Hinweise und Anreize für Um- und Spazierwege auf dem Campus, z. B. Beschilderungen und Markierungen bzw. über eine Campus-Navigations-App – Verbesserung der Sitz‑, Steh- und Bewegungsmöglichkeiten in der Bibliothek – Motivierende Sprüche an Fahrstühlen mit Verweis zur Treppe – Digitale Bewegungspartnerbörse
**Monitoring und Evaluation der Maßnahmen**	Standardisierte Erhebung zur Wahrnehmung, Nutzung und Wirkung der bewegungsfördernden Maßnahmen Erhebung unter Studierenden, die… *– die aktive Pause in der Vorlesung mitgemacht haben* *– die Active-Office-Systeme genutzt hatten* *– sich in Gebäuden aufhielten, an denen die Sprüche angebracht wurden* Semistandardisierte Interviews mit den Teilnehmenden der Planungsgruppen	Hohe Akzeptanz der Maßnahmen unter Studierenden Sichtbarkeit von und Bewusstsein zu Bewegung am Campus wurden laut Interviews verbessert

## Diskussion

### Wichtigste Ergebnisse

Über Methodenmix wurden vielfältige Einflussfaktoren auf das Bewegungsverhalten von Studierenden erfasst. Sie betrafen vorwiegend die Campusstruktur und den Zugang zu Angeboten. Auf der einen Seite scheinen Grünflächen, Fußwege sowie die Innenräume für Bewegung gut nutzbar; andererseits fehlen niedrigschwellige Angebote, wie ausleihbare Sportgeräte oder eine aktive Pausengestaltung. Hinzu kam das als soziale Norm wahrgenommene Verständnis, dass Lernen im Sitzen geschehen müsse und sitzendes Verhalten in Bibliotheken und Vorlesungsräumen unvermeidlich sei.

Basierend auf diesen Erkenntnissen erarbeiteten lokale Planungsgruppen verschiedene Ansätze für aktive Bewegungspausen in den Lehrveranstaltungen, für aktivierende Möbel mit Sitz‑/Stehmöglichkeit, Bewegungshinweise im Außengelände sowie für motivierende Bewegungsanreize. Die entwickelten Maßnahmen sind überwiegend auf der Verhältnisebene angesiedelt, indem veränderte physische und soziale Rahmenbedingungen die Alltagsbewegung erleichtern sollen. Zugleich enthalten sie auch verhaltensorientierte motivationale Ansätze.

Orientierende Evaluationen der Maßnahmen ergaben eine hohe Akzeptanz unter Studierenden, eine Reflexion über das eigene Bewegungsverhalten und die Wahrnehmung von gesteigerter Alltagsbewegung, insbesondere durch die neue Möblierung in der Bibliothek und die aktiven Pausen. Die Covid-19-Pandemie erschwerte bzw. verzögerte im Lauf des Projektes die Umsetzung von Maßnahmen sowie die Evaluation des gesamten Vorhabens hinsichtlich der Auswirkungen auf das Bewegungsverhalten.

### Implikationen für Politik und Praxis und Vergleich mit anderen Studien

Die detaillierte Erhebung von Barrieren und Förderfaktoren konnte wesentliche Ansatzpunkte für bewegungsfördernde Maßnahmen an den Hochschulen identifizieren. Die qualitativen Methoden (Experteninterviews, Photovoice-Ansatz) ermöglichten ein tiefes Verständnis darüber, wie bestimmte Campusstrukturen, eingespielte Abläufe und vorherrschende Normen die Alltagsbewegung von Studierenden beeinflussen.

Die COM-B-Methode half bei der Strukturierung der vielen identifizierten Einflussfaktoren. Während die Einteilung der Faktoren nach „capabilities“, „opportunities“ und „motivation“ sinnvoll für deren Strukturierung war, orientierte sich das Vorgehen in den Planungsgruppen eher an den konkreten Räumen bzw. Einrichtungen der Hochschulen: Außengelände, Innenräume und Hochschulsport. Die Planungsgruppen arbeiteten weitestgehend unabhängig voneinander, nutzten aber interessanterweise ähnliche Ansatzpunkte zur Bewegungsförderung: Unterbrechung der langen Sitzzeiten in Lehrveranstaltungen durch Bewegungspausen, mehr bewegliche Flexibilität beim Mobiliar, z. B. in Bibliotheken, sowie Ausweisung attraktiver Gehwege auf dem Campus.

Die Ansätze wurden allerdings an jeder Hochschule unterschiedlich ausgestaltet, indem sie an die lokalen Gegebenheiten bzw. Ressourcen angepasst wurden. So konnte eine Universität eine existierende Campus-Navigations-App nutzen, um auf Spazierwege hinzuweisen, während an der anderen Universität eine neue Wegemarkierung mit einem existierenden Trimm-dich-Pfad zusammengelegt wurde. Ähnliches galt für die Bewegungspausen, für die eine Universität an die etablierte Zusammenarbeit mit Fitnesstrainern und Fitnesstrainerinnen anknüpfen konnte; an der anderen Universität wurden stattdessen Videos entwickelt, um unabhängig von der Verfügbarkeit von Personal zu sein. Das zeigt, dass die partizipative Planung im Setting wichtig ist, um passgenaue Maßnahmen zu entwickeln, die auch die bestehenden Strukturen und Vorteile einer Hochschule nutzen.

Die Interventionen sind in Einklang mit internationalen Studien zur Bewegungsförderung. So wurden auch an anderen Hochschulen aktivierende Sitzmöbel eingeführt und untersucht, zum Beispiel Stehpultaufsätze oder tragbare Pedaltrainer. Derartige Maßnahmen wurden von Studierenden überwiegend positiv angenommen [27, 28] und erhöhten auch die Bewegungszeiten der Studierenden [29]. Die meisten Studien beziehen sich allerdings auf aktivierende Sitzmöbel in Seminarräumen bzw. deren Nutzung während der Lehrveranstaltung, nur wenige auf die Bibliothek [29]. Umfassendere Daten zur Nutzung und Wirksamkeit aktiver Arbeitsstationen in Bibliotheken fehlen.

Ebenso gibt es Hinweise, dass aktive Pausen in Lehrveranstaltungen positive Auswirkungen bei Studierenden haben [29]. Allerdings fehlen fundierte Evaluationen, auch zu längerfristigen Effekten auf Bewegung. Zudem ist eine Wirksamkeit eher zu erwarten, wenn die Sitzunterbrechungen häufig und regelmäßig stattfinden, d. h. in allen Lehrveranstaltungen nach einem gewissen Zeitintervall (z. B. immer nach 20 min oder 45 min). Ein derart flächendeckender Einsatz wurde bei „Smart Moving“ nicht erreicht, gleichwohl „Dissemination der Bewegungsvideos“ ein Thema war, das die Planungsgruppe an der UR zukünftig angehen wollte.

Die Wirksamkeit von Sprüchen an den Wänden von öffentlichen Gebäuden, Arbeitsstätten bzw. Universitäten, die zur Treppennutzung motivieren sollen, ist bereits wiederholt beschrieben [30]. Auch eine deutsche Studie konnte zeigen, dass partizipativ durch Studierende entwickelte Sprüche, die neben Aufzügen einer Universität angebracht wurden, die Nutzung von Treppen erhöhte [31]. Auch in unserer Studie erklärten 11 % der vor Ort befragten Studierenden, wegen des Schriftzugs an diesem Tag die Treppe zu nehmen oder zumindest darüber nachzudenken. Allerdings müssen bei solchen motivierenden Hinweisen auch mögliche Wear-out-Effekte berücksichtigt werden [32], sodass regelmäßig z. B. Aktionen zur Erinnerung oder neue Impulse für Bewegung eingesetzt werden sollten.

Es gibt bislang wenig Beispiele in der Literatur, die – analog zum bei „Smart Moving“ verwendeten WHO-Ansatz Tailoring Health Programmes – für das Thema „Bewegungsverhalten von Studierenden am Campus“ den gesamten Zyklus von Erhebung der BCI, partizipativer Planung von gesundheitsförderlichen Maßnahmen sowie Umsetzung und Evaluation beschreiben. Die größte Übereinstimmung zu „Smart Moving“ findet sich im „PEAK“-Programm, das an einer australischen Universität basierend auf dem COM-B-Modell durchgeführt wurde [14]. Brown et al. berichten hierzu, dass nach Festlegung des Zielverhaltens (regelmäßiges Sporttreiben bei Studierenden) zunächst Interviews und Fokusgruppen mit Studierenden und Mitarbeitenden zu Barrieren und Bedarfen stattfanden. Die identifizierten Barrieren stimmen teilweise mit den bei „Smart Moving“ gewonnenen Erkenntnissen überein (Fehlen von leicht zugänglichen Bewegungsangeboten auf dem Hochschulgelände, Scham beim Sporttreiben vor anderen), sind aber ansonsten weniger konkret auf die örtliche Struktur und Lernbedingungen bezogen. Ursächlich dafür ist auch, dass es bei Brown et al. primär um Sporttreiben und weniger um Alltagsbewegung geht. Maßnahmen wurden bei „PEAK“ ebenfalls partizipativ entwickelt und umfassten neue kostenfreie Sportangebote, wöchentliche motivationale Videos, eine soziale Unterstützungsgruppe über soziale Medien sowie die Gründung einer Sportgruppe [14].

In „Smart Moving“ hat das Photovoice-Projekt nicht nur Erkenntnisse zu den bewegungsbezogenen Rahmenbedingungen an den Campus generiert, sondern konnte auch das Bewusstsein der Teilnehmenden für die Themen Bewegung und Verhältnisprävention erhöhen; einige Photovoice-Teilnehmende wurden daher auch Mitglieder der Planungsgruppen. Die Methode eignete sich also sehr gut dafür, Datenerhebung und Maßnahmenplanung zu verknüpfen.

Weiterhin zeigte sich, dass die Mehrheit der gewählten Maßnahmen zwar vor Ort an den Hochschulen umgesetzt wurde, aber auch digitale Ansätze zur Bewegungsförderung mit integriert wurden. Diese hatten jeweils einen lokalen Bezug, z. B. die Aufnahme von Spazierwegen in eine Uni-Navigations-App oder die Online-basierte Bewegungspartnerbörse. Die über YouTube verfügbaren Videos zur Bewegten Pause wurden im örtlichen Hörsaal gefilmt. Diese Beispiele zeigen, wie moderne Medien in der Prävention genutzt werden können, ohne die Anbindung an die „Offline-Welt“ zu verlieren.

Erfreulicherweise gelang an beiden Universitäten eine gewisse Nachhaltigkeit der Strukturen und Maßnahmen. Die Bewegungspausen werden jeweils durch das universitäre bzw. studentische Gesundheitsmanagement (SGM) weitergeführt und ausgebaut. Weiterhin ist die Sport- und Bewegungspartnerbörse Teil des Allgemeinen Hochschulsports an der UBT und das bewegungsfördernde Sitz- und Stehmobiliar ist an den Bibliotheken dauerhaft etabliert, ebenso die Aktivpfade an beiden Campus. Die Studie verdeutlicht, dass die Anbindung einer projektbasierten Planungsgruppe an feste Strukturen eines Settings wichtig ist, um Nachhaltigkeit zu ermöglichen. Im Fall von „Smart Moving“ hat sich das SGM bewährt, das in Anlehnung an das betriebliche Gesundheitsmanagement konzipiert und an vielen deutschen Hochschulen implementiert wurde. SGM soll gesundheitsbezogene Prozesse im Zusammenhang mit Studierenden an einer Hochschule bündeln und dabei Studierende partizipativ einbinden [7].

„Smart Moving“ zeigt am Beispiel Bewegung, dass das Konzept Tailoring Health Programmes helfen kann, partizipative, bedarfsgerechte Mehrebenen-Interventionen erfolgreich in einem Setting zu implementieren, basierend auf verhaltensbezogenen Erkenntnissen. Im Sinne eines breiteren Public-Health-Ansatzes wäre es zielführend, den Ansatz auf weitere Gesundheitsthemen auszuweiten. Im Setting Hochschule böte sich dabei das Thema psychische Gesundheit von Studierenden an; hier zeigen internationale Studien, dass – ähnlich wie bei Bewegung – die Rahmenbedingungen am Campus eine wichtige Rolle spielen, zudem niedrigschwellige Angebote fehlen und Barrieren existieren, um professionelle Hilfe aufzusuchen [33, 34]. Es ist gut vorstellbar, dass es zielführend sein kann, Campus-spezifische Erkenntnisse zu sammeln und in Planungsgruppen lokal angepasste Maßnahmen zur Förderung psychischer Gesundheit umzusetzen. Weitere relevante Public-Health-Themen für Studierende könnten gesunde Ernährung oder auch Alkoholkonsum [35, 36] sein.

### Stärken und Limitationen

Die vorliegende Studie zeigt am Beispiel zweier Hochschulen, wie bewegungsfördernde Maßnahmen basierend auf dem BCI-Ansatz Schritt für Schritt als partizipativer Ansatz geplant und umgesetzt werden können. Die detaillierte Beschreibung der Prozesse und Maßnahmen ermöglicht, ähnliche Projekte an anderen Hochschulen durchzuführen.

Bei beiden Hochschulen handelt es sich um sogenannte Campus-Universitäten mit einem großen Außengelände und vielen Grünflächen. Daher lassen sich nicht alle Ansätze auf kompaktere bzw. in Innenstädten integrierte Hochschulen übertragen. Eine weitere Limitation ist, dass die Auswirkungen auf das Bewegungsverhalten der Studierenden nicht evaluiert wurden. Die geplante Pre-Post-Erhebung ließ sich aufgrund der Covid-19-Pandemie nicht realisieren. Die eingesetzten Erhebungen zu den Einzelmaßnahmen ermöglichten nur orientierende Erkenntnisse über Akzeptanz und mögliche Verhaltensintentionen.

## Fazit

Der BCI-Ansatz, wie ihn die WHO vorschlägt, ermöglicht die bedarfsgerechte Planung und Umsetzung von bewegungsfördernden Maßnahmen. Eine Anpassung an die lokalen Gegebenheiten schafft Synergien mit bestehenden Aktivitäten und Ressourcen. Entscheidend ist zudem, interessierte Vertreter und Vertreterinnen aus verschiedenen Bereichen der Hochschule eng einzubinden, neben Studierenden und Lehrenden z. B. auch Mitarbeitende aus der Bibliothek. So können bewegungsfördernde Ansätze leichter und nachhaltiger in die Hochschulabläufe und -strukturen integriert werden. Studien sollten die langfristigen Auswirkungen von multidimensionalen Ansätzen auf das Bewegungsverhalten von Studierenden untersuchen. Zudem machen die Ergebnisse Mut, den Ansatz auch auf andere Public-Health-relevante Themen auszuweiten, z. B. Alkoholkonsum oder mentale Gesundheit.

## Data Availability

Die während der vorliegenden Studie erzeugten und/oder analysierten Datensätze sind teilweise aufgrund von schwer umsetzbarer Anonymisierung (z. B. Interviews mit Personen der Universitätsverwaltungen) nicht öffentlich zugänglich, können aber auf begründete Anfrage bei den entsprechenden Autorinnen und Autoren angefordert werden.

## References

[1] Lee IM, Shiroma EJ, Lobelo F et al (2012) Effect of physical inactivity on major non-communicable diseases worldwide: an analysis of burden of disease and life expectancy. Lancet 380:219–229. https://doi.org/10.1016/S0140–6736(12)61031‑9 22818936 10.1016/S0140-6736(12)61031-9 PMC3645500

[2] Posadzki P, Pieper D, Bajpai R et al (2020) Exercise/physical activity and health outcomes: an overview of Cochrane systematic reviews. BMC Public Health 20:1724. 10.1186/s12889-020-09855-3 33198717 10.1186/s12889-020-09855-3 PMC7670795

[3] Saunders TJ, McIsaac T, Douillette K et al (2020) Sedentary behaviour and health in adults: an overview of systematic reviews. Appl Physiol Nutr Metab 45:S197–S217. 10.1139/apnm-2020-0272 33054341 10.1139/apnm-2020-0272

[4] Wallmann-Sperlich B, Bucksch J, Hansen S, Schantz P, Froboese I (2013) Sitting time in Germany: an analysis of socio-demographic and environmental correlates. BMC Public Health 13:196. 10.1186/1471-2458-13-196 23497070 10.1186/1471-2458-13-196 PMC3605332

[5] Manz K, Domanska OM, Krug S, Kuhnert R (2022) Wie viel sitzen Erwachsene? Ergebnisse der Studie Gesundheit in Deutschland aktuell (GEDA 2019/2020-EHIS). J Health Monit 7:32–40. 10.25646/1029

[6] Richter A, Schienkiewitz A, Starker A et al (2021) Gesundheitsfördernde Verhaltensweisen bei Erwachsenen in Deutschland – Ergebnisse der Studie GEDA 2019/2020-EHIS. J Health Monit 6:28–48. 10.25646/8460.2

[7] Voermans S, König S, Niemeyer I et al (2019) SGM – Studentisches Gesundheitsmanagement. Handlungsempfehlung zu Theorie und Praxis. Techniker Krankenkasse, Hamburg

[8] Irvine KN, Fisher D, Currie M, Colley K, Warber SL (2024) A Nature-Based Intervention for Promoting Physical Activity in Older Adults: A Qualitative Study Using the COM‑B Model. Int J Environ Res Public Health. 10.3390/ijerph21070843 39063420 10.3390/ijerph21070843 PMC11276442

[9] Michie S, van Stralen MM, West R (2011) The behaviour change wheel: a new method for characterising and designing behaviour change interventions. Implement Sci 6:42. 10.1186/1748-5908-6-42 21513547 10.1186/1748-5908-6-42 PMC3096582

[10] WHO Regional Office for Europe (2023) A guide to tailoring health programmes: using behavioural and cultural insights to tailor health policies, services and communications to the needs and circumstances of people and communities. WHO Regional Office for Europe, Copenhagen

[11] West R, Gould A (2022) Improving health and wellbeing: a guide to using behavioural science in policy and practice. Public Health Wales NHS Trust, Cardiff

[12] Artinger S, Baltes S, Jacobs P, Jarchow C, Petersen M, Schneider AM (2021) Patientensicherheit im Krankenhaus: Gemeinsam für die Infektionsprävention. Untersuchung zur Wirkung eines Programms zur Infektionsprävention durch Partizipation, Feedback und Leadership auf Intensivstationen. Presse- und Informationsamt der Bundesregierung, Berlin

[13] Moore AP, Rivas CA, Stanton-Fay S, Harding S, Goff LM (2019) Designing the Healthy Eating and Active Lifestyles for Diabetes (HEAL-D) self-management and support programme for UK African and Caribbean communities: a culturally tailored, complex intervention under-pinned by behaviour change theory. BMC Public Health 19:1146. 10.1186/s12889-019-7411-z 31429735 10.1186/s12889-019-7411-z PMC6702734

[14] Brown CEB, Richardson K, Halil-Pizzirani B et al (2024) Developing the PEAK mood, mind, and marks program to support university students’ mental and cognitive health through physical exercise: a qualitative study using the Behaviour Change Wheel. BMC Public Health 24:1959. 10.1186/s12889-024-19385-x 39039474 10.1186/s12889-024-19385-x PMC11265317

[15] Ojo SO, Bailey DP, Brierley ML, Hewson DJ, Chater AM (2019) Breaking barriers: using the behavior change wheel to develop a tailored intervention to overcome workplace inhibitors to breaking up sitting time. BMC Public Health 19:1126. 10.1186/s12889-019-7468-8 31420033 10.1186/s12889-019-7468-8 PMC6697980

[16] von Sommoggy J, Rueter J, Curbach J, Helten J, Tittlbach S, Loss J (2020) How Does the Campus Environment Influence Everyday Physical Activity? A Photovoice Study Among Students of Two German Universities. Front Public Health 8:561175. 10.3389/fpubh.2020.561175 33123509 10.3389/fpubh.2020.561175 PMC7571200

[17] Mann D, Helten J, Hoffmann SW et al (2021) Bewegungsfördernde Bibliotheksarbeitsplätze an Hochschulen. Präv Gesundheitsf 16:290–295. 10.1007/s11553-020-00806-9

[18] Helten J (2022) Smart Moving: Die Rolle verschiedener Einflussfaktoren auf das Bewegungs- und Sitzverhalten von Studierenden. Shaker, Düren

[19] Loss J, Betsch C, Ellermann C et al (2025) Grundzüge des Ansatzes der „Behavioural and Cultural Insights (BCI)“ für eine Nutzung in Public Health – Ein Konsenspapier des Netzwerks „Behavioural Science Connect“. Gesundheitswesen 10.1055/a-2528-827610.1055/a-2528-8276 ((Characteristics of the „Behavioural and cultural insights (BCI)“ approach in Public Health—a consensus paper of the network „Behavioural Science Connect“)) 10.1055/a-2528-8276 39880008

[20] Fuchs R, Klaperski S, Gerber M, Seelig H (2015) Messung der Bewegungs- und Sportaktivität mit dem BSA-Fragebogen. Z Gesundheitspsychol 23:60–76

[21] Marshall AL, Miller YD, Burton NW, Brown WJ (2010) Measuring total and domain-specific sitting: a study of reliability and validity. Med Sci Sports Exerc 42:1094–1102. 10.1249/MSS.0b013e3181c5ec18 19997030 10.1249/MSS.0b013e3181c5ec18

[22] Brehm W, Pahmeier I, Tiemann M, Wagner P, Bös K (2014) Psychosoziale Ressourcen. Stärkung von psychosozialen Rressourcen im Fitness- und Gesundheitssport. Deutscher Turner-Bund, Frankfurt/Main

[23] Wang CC, Yi WK, Tao ZW, Carovano K (1998) Photovoice as a Participatory Health Promotion Strategy. Health Promot Int 13:75–86. 10.1093/heapro/13.1.75

[24] Ritchie J, Lewis J, McNaughton Nicholls C (2013) Qualitative Research Practice: A Guide for Social Science Students and Researchers. SAGE Publishing, Thousand Oaks

[25] Michie S, Van Stralen M, West R (2011) The behaviour change wheel: A new method for characterising and designing behaviour change interventions. Implement Sci 6:42. 10.1186/1748-5908-6-42 21513547 10.1186/1748-5908-6-42 PMC3096582

[26] Helten J, Tittlbach S (2024) Bewegt studieren – studieren bewegt an der Universität Bayreuth. Publ Health Forum 32:127–130

[27] Benzo RM, Gremaud AL, Jerome M, Carr LJ (2016) Learning to Stand: The Acceptability and Feasibility of Introducing Standing Desks into College Classrooms. Int J Environ Res Public Health. 10.3390/ijerph13080823 27537901 10.3390/ijerph13080823 PMC4997509

[28] Tardif BC, Cantin M, Senecal S et al (2018) Implementation of Active Workstations in University Libraries—A Comparison of Portable Pedal Exercise Machines and Standing Desks. Int J Environ Res Public Health. 10.3390/ijerph15061242 29895760 10.3390/ijerph15061242 PMC6024930

[29] Lynch J, O’Donoghue G, Peiris CL (2022) Classroom Movement Breaks and Physically Active Learning Are Feasible, Reduce Sedentary Behaviour and Fatigue, and May Increase Focus in University Students: A Systematic Review and Meta-Analysis. Int J Environ Res Public Health. 10.3390/ijerph19137775 35805432 10.3390/ijerph19137775 PMC9265656

[30] Bellicha A, Kieusseian A, Fontvieille AM, Tataranni A, Charreire H, Oppert JM (2015) Stair-use interventions in worksites and public settings—a systematic review of effectiveness and external validity. Prev Med 70:3–13. 10.1016/j.ypmed.2014.11.001 25449692 10.1016/j.ypmed.2014.11.001

[31] Bachert P, Hildebrand C, Erley N et al (2022) Students on stairs: a participatory approach using decisional cues in the form of motivational signs to promote stair use. J Am Coll Health 70:2152–2158. 10.1080/07448481.2020.1845704 33427112 10.1080/07448481.2020.1845704

[32] Harnischmacher J, Merkl LM, Germelmann CC (2024) Nudging Physical Distance During COVID-19 : Short-Term and Long-Term Wear-Out Effects of Nudges in a Retail Setting. In: Jeseo V, Allen J (Hrsg) 2023 AMS Annual Conference Welcome to The New Normal: Life After The Chaos. Springer, New Orleans, USA, S 197–207

[33] Hyseni Duraku Z, Davis H, Hamiti E (2023) Mental health, study skills, social support, and barriers to seeking psychological help among university students: a call for mental health support in higher education. Front Public Health 11:1220614. 10.3389/fpubh.2023.1220614 37920583 10.3389/fpubh.2023.1220614 PMC10619655

[34] Moghimi E, Stephenson C, Gutierrez G et al (2023) Mental health challenges, treatment experiences, and care needs of post-secondary students: a cross-sectional mixed-methods study. BMC Public Health 23:655. 10.1186/s12889-023-15452-x 37020282 10.1186/s12889-023-15452-x PMC10076091

[35] Cimini MD, Martin JL (2022) Promoting uptake of efficacious brief alcohol interventions for young adults within institutions of higher education: Challenges and opportunities. Psychol Addict Behav 36:741–747. 10.1037/adb0000857 35797167 10.1037/adb0000857

[36] Wolfson M, Champion H, McCoy TP et al (2012) Impact of a randomized campus/community trial to prevent high-risk drinking among college students. Alcohol Clin Exp Res 36:1767–1778. 10.1111/j.1530-0277.2012.01786.x 22823091 10.1111/j.1530-0277.2012.01786.x PMC3752385

